# (*E*)-1-(5-Bromo-2-hy­droxy­phen­yl)-3-[4-(dimethyl­amino)­phen­yl]prop-2-en-1-one

**DOI:** 10.1107/S1600536812016327

**Published:** 2012-04-21

**Authors:** Guang-Bing Li, Lu Li, Guo-Xi Wang

**Affiliations:** a Department of Chemical & Environmental Engineering, Anyang Institute of Technology, Anyang 455000, People’s Republic of China

## Abstract

In the title compound, C_17_H_16_BrNO_2_, the two benzene rings make a dihedral angle of 7.4 (3)°; the hy­droxy group links to the carbonyl group *via* an intra­molecular O—H⋯O hydrogen bond. In the crystal, weak C—H⋯O inter­actions link the mol­ecules into a supra­molecular chain running along the *c* axis.

## Related literature

For related compounds and structures, see: Dai & Chen (2011 ▶); Xu *et al.* (2011 ▶); Fu *et al.* (2011 ▶); Zheng *et al.* (2011 ▶).
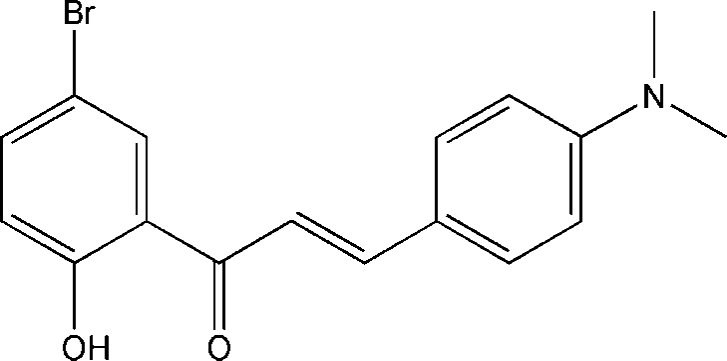


## Experimental

### 

#### Crystal data

C_17_H_16_BrNO_2_
*M* *_r_* = 346.22
Monoclinic, 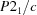

*a* = 15.1905 (5) Å
*b* = 5.4501 (2) Å
*c* = 19.7569 (9) Å
β = 106.00 (2)°
*V* = 1572.34 (11) Å^3^
*Z* = 4
Mo *K*α radiation
μ = 2.62 mm^−1^
*T* = 123 K
0.30 × 0.05 × 0.05 mm

#### Data collection

Rigaku Mercury2 diffractometer
Absorption correction: multi-scan (*CrystalClear*; Rigaku, 2005 ▶) *T*_min_ = 0.910, *T*_max_ = 1.000
8044 measured reflections
2736 independent reflections
1538 reflections with *I* > 2σ(*I*)
*R*_int_ = 0.075

#### Refinement

*R*[*F* ^2^ > 2σ(*F* ^2^)] = 0.089
*wR*(*F* ^2^) = 0.287
*S* = 1.09
2736 reflections
193 parameters
1 restraint
H-atom parameters constrained
Δρ_max_ = 1.58 e Å^−3^
Δρ_min_ = −1.11 e Å^−3^

### 

Data collection: *CrystalClear* (Rigaku, 2005 ▶); cell refinement: *CrystalClear*; data reduction: *CrystalClear*; program(s) used to solve structure: *SHELXTL* (Sheldrick, 2008 ▶); program(s) used to refine structure: *SHELXTL*; molecular graphics: *SHELXTL*; software used to prepare material for publication: *SHELXTL*.

## Supplementary Material

Crystal structure: contains datablock(s) I, global. DOI: 10.1107/S1600536812016327/xu5515sup1.cif

Structure factors: contains datablock(s) I. DOI: 10.1107/S1600536812016327/xu5515Isup2.hkl

Supplementary material file. DOI: 10.1107/S1600536812016327/xu5515Isup3.cml

Additional supplementary materials:  crystallographic information; 3D view; checkCIF report

## Figures and Tables

**Table 1:** Hydrogen-bond geometry (Å, °)

*D*—H⋯*A*	*D*—H	H⋯*A*	*D*⋯*A*	*D*—H⋯*A*
O2—H2⋯O1	0.82	1.82	2.543 (7)	146
C17—H17*A*⋯O2^i^	0.98	2.55	3.503 (10)	164

Symmetry code: (i) 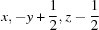.

**Fig. 1.**
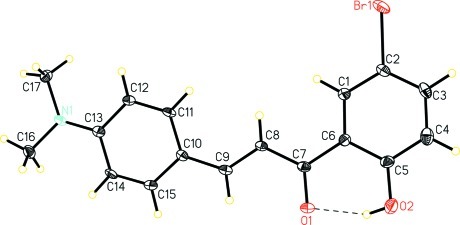
Molecular view of the title compound with the atomic numbering scheme. Displacement ellipsoids are drawn at the 30% probability level.

## supplementary crystallographic information

### Comment

Organic amine derivatives are becoming increasingly important as new molecule-based crystalline materials with the potential optimal physical properties (Dai & Chen, 2011; Xu *et al.*, 2011). In addition, the amino compounds have found a wide range of applications in coordination chemistry as ligands to construct novel crystal structures (Fu *et al.*, 2011; Zheng, 2011). Herein, we report the crystal structure of the title compound, (E)-1-(5-bromo-2-hydroxyphenyl)-3-(4-(dimethylamino)phenyl)prop-2-en-1-one.

The asymmetric unit is composed of one whole amine molecule. The two benzene rings are nearly coplanar and twisted from each other by a dihedral of 7.4 (3)°. The hydroxy H2 atom was involved in intramolecular O—H···O hydrogen bonds interaction (Table 1 and Fig. 1).

### Experimental

The title compound (2.0 mmol) was solved in 50 mL methanol. Then the solution was evaporated slowly in the air. Red block crystals suitable for X-ray analysis were obtained after one week.

### Refinement

All H atoms attached to C atoms were fixed geometrically and treated as riding with C—H = 0.95 Å (aromatic) and C—H = 0.98 Å (methyl) with *U*_iso_(H) = 1.2*U*_eq_(C) and *U*_iso_(H) = 1.5*U*_eq_(methyl). H atoms bonded to O atom was located in difference Fourier map and restrained with the H—O = 0.82 (2) Å. In the last stage of refinement they were treated as riding on the O atom with *U*_iso_(H) = 1.5*U*_eq_(O).

### Crystal data

**Table:**

C_17_H_16_BrNO_2_	*F*(000) = 704
*M**_r_* = 346.22	*D*_x_ = 1.463 Mg m^−^^3^
Monoclinic, *P*2_1_/*c*	Mo *K*α radiation, λ = 0.71073 Å
Hall symbol: -P 2ybc	Cell parameters from 2736 reflections
*a* = 15.1905 (5) Å	θ = 2.2–27.5°
*b* = 5.4501 (2) Å	µ = 2.62 mm^−^^1^
*c* = 19.7569 (9) Å	*T* = 123 K
β = 106.00 (2)°	Needle, red
*V* = 1572.34 (11) Å^3^	0.30 × 0.05 × 0.05 mm
*Z* = 4	

### Data collection

**Table:**

Rigaku Mercury2 diffractometer	2736 independent reflections
Radiation source: fine-focus sealed tube	1538 reflections with *I* > 2σ(*I*)
Graphite monochromator	*R*_int_ = 0.075
Detector resolution: 13.6612 pixels mm^-1^	θ_max_ = 25.0°, θ_min_ = 2.2°
CCD profile fitting scans	*h* = −18→17
Absorption correction: multi-scan (*CrystalClear*; Rigaku, 2005)	*k* = −6→6
*T*_min_ = 0.910, *T*_max_ = 1.000	*l* = −23→23
8044 measured reflections	

### Refinement

**Table:**

Refinement on *F*^2^	Secondary atom site location: difference Fourier map
Least-squares matrix: full	Hydrogen site location: inferred from neighbouring sites
*R*[*F*^2^ > 2σ(*F*^2^)] = 0.089	H-atom parameters constrained
*wR*(*F*^2^) = 0.287	*w* = 1/[σ^2^(*F*_o_^2^) + (0.141*P*)^2^ + 0.6743*P*] where *P* = (*F*_o_^2^ + 2*F*_c_^2^)/3
*S* = 1.09	(Δ/σ)_max_ < 0.001
2736 reflections	Δρ_max_ = 1.58 e Å^−^^3^
193 parameters	Δρ_min_ = −1.11 e Å^−^^3^
1 restraint	Extinction correction: *SHELXTL* (Sheldrick, 2008), Fc^*^=kFc[1+0.001xFc^2^λ^3^/sin(2θ)]^-1/4^
Primary atom site location: structure-invariant direct methods	Extinction coefficient: 0.002

### Special details

**Table:**

Geometry. All esds (except the esd in the dihedral angle between two l.s. planes) are estimated using the full covariance matrix. The cell esds are taken into account individually in the estimation of esds in distances, angles and torsion angles; correlations between esds in cell parameters are only used when they are defined by crystal symmetry. An approximate (isotropic) treatment of cell esds is used for estimating esds involving l.s. planes.
Refinement. Refinement of F^2^ against ALL reflections. The weighted R-factor wR and goodness of fit S are based on F^2^, conventional R-factors R are based on F, with F set to zero for negative F^2^. The threshold expression of F^2^ > 2sigma(F^2^) is used only for calculating R-factors(gt) etc. and is not relevant to the choice of reflections for refinement. R-factors based on F^2^ are statistically about twice as large as those based on F, and R- factors based on ALL data will be even larger.

### Fractional atomic coordinates and isotropic or equivalent isotropic displacement parameters (Å^2^)

**Table:**

	*x*	*y*	*z*	*U*_iso_*/*U*_eq_	
Br1	1.08032 (7)	−0.2556 (2)	0.57680 (7)	0.1050 (7)	
O1	0.6988 (3)	0.0521 (10)	0.6480 (2)	0.0469 (13)	
C7	0.7599 (4)	0.0665 (12)	0.6153 (3)	0.0329 (15)	
N1	0.5880 (3)	1.1541 (11)	0.3308 (3)	0.0355 (14)	
O2	0.7796 (4)	−0.3021 (10)	0.7239 (3)	0.0600 (16)	
H2	0.7357	−0.2242	0.7002	0.090*	
C10	0.6627 (4)	0.6074 (12)	0.4911 (3)	0.0307 (14)	
C9	0.6821 (4)	0.4141 (13)	0.5444 (3)	0.0331 (15)	
H9A	0.6394	0.3991	0.5714	0.040*	
C14	0.5541 (4)	0.9164 (12)	0.4255 (3)	0.0339 (15)	
H14A	0.4969	0.9979	0.4183	0.041*	
C13	0.6124 (4)	0.9790 (12)	0.3830 (3)	0.0331 (15)	
C5	0.8429 (5)	−0.2911 (12)	0.6878 (4)	0.0386 (17)	
C12	0.6988 (4)	0.8490 (12)	0.3966 (3)	0.0362 (16)	
H12A	0.7404	0.8867	0.3699	0.043*	
C1	0.9105 (4)	−0.1047 (14)	0.6015 (3)	0.0412 (17)	
H1A	0.9106	0.0160	0.5669	0.049*	
C6	0.8391 (4)	−0.1080 (12)	0.6344 (3)	0.0307 (14)	
C2	0.9802 (5)	−0.2758 (13)	0.6193 (4)	0.0446 (19)	
C15	0.5789 (5)	0.7398 (12)	0.4769 (4)	0.0342 (16)	
H15A	0.5380	0.7044	0.5043	0.041*	
C11	0.7207 (4)	0.6699 (12)	0.4483 (3)	0.0364 (16)	
H11A	0.7772	0.5849	0.4555	0.044*	
C4	0.9150 (5)	−0.4652 (14)	0.7037 (4)	0.0483 (19)	
H4A	0.9168	−0.5864	0.7387	0.058*	
C8	0.7530 (4)	0.2529 (11)	0.5605 (3)	0.0294 (15)	
H8A	0.7987	0.2610	0.5360	0.035*	
C16	0.5060 (5)	1.3053 (13)	0.3242 (4)	0.0429 (18)	
H16A	0.4515	1.2004	0.3123	0.064*	
H16B	0.5012	1.4277	0.2871	0.064*	
H16C	0.5105	1.3887	0.3690	0.064*	
C3	0.9833 (4)	−0.4609 (14)	0.6688 (4)	0.0438 (18)	
H3A	1.0306	−0.5806	0.6784	0.053*	
C17	0.6536 (6)	1.2333 (13)	0.2900 (4)	0.0441 (18)	
H17A	0.6841	1.0888	0.2775	0.066*	
H17B	0.6994	1.3444	0.3190	0.066*	
H17C	0.6197	1.3183	0.2470	0.066*	

### Atomic displacement parameters (Å^2^)

**Table:**

	*U*^11^	*U*^22^	*U*^33^	*U*^12^	*U*^13^	*U*^23^
Br1	0.0628 (9)	0.1585 (14)	0.1133 (11)	0.0652 (8)	0.0570 (8)	0.0639 (8)
O1	0.038 (3)	0.055 (3)	0.057 (3)	0.006 (2)	0.029 (2)	0.010 (2)
C7	0.024 (3)	0.036 (4)	0.041 (4)	−0.005 (3)	0.012 (3)	−0.004 (3)
N1	0.029 (3)	0.041 (3)	0.039 (3)	0.008 (2)	0.014 (3)	−0.003 (2)
O2	0.049 (4)	0.063 (4)	0.069 (4)	0.005 (3)	0.020 (3)	0.029 (3)
C10	0.026 (3)	0.032 (3)	0.034 (3)	0.002 (3)	0.008 (3)	−0.004 (3)
C9	0.024 (3)	0.043 (4)	0.034 (3)	0.000 (3)	0.011 (3)	−0.006 (3)
C14	0.026 (3)	0.033 (3)	0.047 (4)	0.006 (3)	0.017 (3)	−0.009 (3)
C13	0.030 (3)	0.036 (4)	0.033 (3)	−0.002 (3)	0.008 (3)	−0.008 (3)
C5	0.034 (4)	0.040 (4)	0.045 (4)	−0.007 (3)	0.016 (3)	0.001 (3)
C12	0.027 (4)	0.040 (4)	0.045 (4)	0.000 (3)	0.016 (3)	0.002 (3)
C1	0.038 (4)	0.050 (4)	0.039 (4)	0.010 (3)	0.016 (3)	0.012 (3)
C6	0.026 (3)	0.036 (4)	0.027 (3)	−0.003 (3)	0.003 (3)	0.001 (3)
C2	0.032 (4)	0.053 (5)	0.052 (5)	0.011 (3)	0.015 (4)	0.005 (3)
C15	0.028 (4)	0.041 (4)	0.037 (4)	−0.003 (3)	0.015 (3)	−0.006 (3)
C11	0.024 (3)	0.046 (4)	0.043 (4)	0.006 (3)	0.016 (3)	−0.002 (3)
C4	0.044 (4)	0.044 (4)	0.046 (4)	−0.004 (3)	−0.005 (4)	0.004 (3)
C8	0.021 (3)	0.035 (3)	0.033 (4)	−0.001 (3)	0.009 (3)	−0.003 (3)
C16	0.046 (5)	0.044 (4)	0.038 (4)	0.016 (3)	0.011 (3)	0.006 (3)
C3	0.030 (4)	0.043 (4)	0.049 (4)	0.008 (3)	−0.003 (3)	−0.001 (3)
C17	0.054 (5)	0.038 (4)	0.046 (4)	0.000 (3)	0.025 (4)	0.005 (3)

### Geometric parameters (Å, º)

**Table:**

Br1—C2	1.933 (8)	C5—C6	1.441 (9)
O1—C7	1.271 (7)	C12—C11	1.386 (9)
C7—C8	1.468 (9)	C12—H12A	0.9500
C7—C6	1.499 (9)	C1—C2	1.382 (9)
N1—C13	1.380 (8)	C1—C6	1.409 (9)
N1—C16	1.469 (8)	C1—H1A	0.9500
N1—C17	1.506 (9)	C2—C3	1.396 (10)
O2—C5	1.347 (9)	C15—H15A	0.9500
O2—H2	0.8200	C11—H11A	0.9500
C10—C11	1.421 (9)	C4—C3	1.397 (9)
C10—C15	1.423 (9)	C4—H4A	0.9500
C10—C9	1.461 (9)	C8—H8A	0.9500
C9—C8	1.358 (9)	C16—H16A	0.9800
C9—H9A	0.9500	C16—H16B	0.9800
C14—C15	1.374 (9)	C16—H16C	0.9800
C14—C13	1.419 (8)	C3—H3A	0.9500
C14—H14A	0.9500	C17—H17A	0.9800
C13—C12	1.450 (9)	C17—H17B	0.9800
C5—C4	1.417 (10)	C17—H17C	0.9800
			
O1—C7—C8	120.3 (6)	C1—C2—C3	122.8 (7)
O1—C7—C6	118.8 (6)	C1—C2—Br1	119.4 (6)
C8—C7—C6	120.9 (5)	C3—C2—Br1	117.8 (5)
C13—N1—C16	120.0 (5)	C14—C15—C10	123.3 (6)
C13—N1—C17	121.2 (5)	C14—C15—H15A	118.3
C16—N1—C17	117.5 (6)	C10—C15—H15A	118.3
C5—O2—H2	105.1	C12—C11—C10	123.1 (6)
C11—C10—C15	115.4 (6)	C12—C11—H11A	118.5
C11—C10—C9	124.6 (6)	C10—C11—H11A	118.5
C15—C10—C9	120.0 (6)	C3—C4—C5	120.9 (7)
C8—C9—C10	128.6 (6)	C3—C4—H4A	119.6
C8—C9—H9A	115.7	C5—C4—H4A	119.6
C10—C9—H9A	115.7	C9—C8—C7	120.7 (6)
C15—C14—C13	121.1 (6)	C9—C8—H8A	119.7
C15—C14—H14A	119.5	C7—C8—H8A	119.7
C13—C14—H14A	119.5	N1—C16—H16A	109.5
N1—C13—C14	121.7 (6)	N1—C16—H16B	109.5
N1—C13—C12	121.2 (5)	H16A—C16—H16B	109.5
C14—C13—C12	117.0 (6)	N1—C16—H16C	109.5
O2—C5—C4	118.2 (6)	H16A—C16—H16C	109.5
O2—C5—C6	121.8 (6)	H16B—C16—H16C	109.5
C4—C5—C6	119.9 (7)	C2—C3—C4	118.1 (6)
C11—C12—C13	120.1 (6)	C2—C3—H3A	120.9
C11—C12—H12A	119.9	C4—C3—H3A	120.9
C13—C12—H12A	119.9	N1—C17—H17A	109.5
C2—C1—C6	120.5 (6)	N1—C17—H17B	109.5
C2—C1—H1A	119.8	H17A—C17—H17B	109.5
C6—C1—H1A	119.8	N1—C17—H17C	109.5
C1—C6—C5	117.7 (6)	H17A—C17—H17C	109.5
C1—C6—C7	122.8 (6)	H17B—C17—H17C	109.5
C5—C6—C7	119.4 (6)		

### Hydrogen-bond geometry (Å, º)

**Table:**

*D*—H···*A*	*D*—H	H···*A*	*D*···*A*	*D*—H···*A*
O2—H2···O1	0.82	1.82	2.543 (7)	146
C17—H17*A*···O2^i^	0.98	2.55	3.503 (10)	164

Symmetry code: (i) *x*, −*y*+1/2, *z*−1/2.
